# Relationship conflict and partner violence by UK military personnel following return from deployment in Iraq and Afghanistan

**DOI:** 10.1007/s00127-022-02317-8

**Published:** 2022-06-05

**Authors:** Rebecca Lane, Roxanna Short, Margaret Jones, Lisa Hull, Louise M. Howard, Nicola T. Fear, Deirdre MacManus

**Affiliations:** 1 Department of Forensic and Neurodevelopmental Sciences, Institute of Psychiatry, Psychology and Neuroscience, King’s College London, 16 De Crespigny Park, Camberwell, London, SE5 8AB UK; 2 King’s Centre for Military Health Research, Weston Education Centre, King’s College London, 10 Cutcombe Road, London, SE5 9RJ UK; 3 Section of Women’s Mental Health, Institute of Psychiatry, Psychology and Neuroscience, King’s College London, Dr Crespigny Park, London, SE5 8AF UK

**Keywords:** Military personnel, Relationships, Military trauma, Deployment, Domestic abuse, Partner violence

## Abstract

**Purpose:**

Risk of violence by UK military personnel, both towards non-family and family, has been found to be higher post-deployment. However, no UK research to date has attempted to examine relationship conflict and intimate partner violence (IPV) in this period. This study estimated the prevalence of and risk factors for post-deployment relationship conflict and partner violence in UK military personnel.

**Methods:**

We utilised data on military personnel who had deployed to Iraq and/or Afghanistan (*n* = 5437), drawn from a large cohort study into the health and well-being of UK military personnel.

**Results:**

34.7% reported relationship conflict (arguing with partner) and 3.4% reported perpetrating physical IPV post-deployment. Males were more likely than females to report relationship conflict. There were similar rates of self-reported physical IPV perpetration among males and females. Among our male sample, factors associated with both relationship conflict and physical IPV perpetration post-deployment included being in the Army compared with the Royal Air Force, higher levels of childhood adversity, higher levels of military trauma exposure and recent mental health and alcohol misuse problems. Being over 40 at time of deployment (vs being under 25) and having deployed in a combat role were also associated with relationship conflict, but not physical IPV perpetration.

**Conclusions:**

Deployment-related variables and mental health and alcohol misuse problems were found to be key factors associated with post-deployment relationship conflict and IPV. Services providing health or welfare support to military personnel must collaborate with mental health services and consider history of deployment, and particularly deployment-related trauma, in their assessments to improve identification and management of intimate partner violence and abuse in military communities.

**Supplementary Information:**

The online version contains supplementary material available at 10.1007/s00127-022-02317-8.

## Introduction

Intimate partner violence and abuse (IPVA), defined by the World Health Organisation as behaviour by a current or ex-partner who causes physical, sexual or psychological harm, including controlling behaviours, is a major global public health problem [1, 2]. It has been marked as a UK Government priority, as evidenced by its recent launch of the Domestic Abuse Act [3]. In March 2020, an estimated 2.3–2.4 million adults reported experiencing domestic abuse in the past year in England and Wales [4]. This figure is likely higher today given the increased incidence reported during the Covid-19 pandemic [5, 6]. Along with associated adverse health effects, including physical injury, mental health and alcohol problems [7, 8], IPVA is estimated to cost £66 billion per annum in England and Wales alone [9]. Exposure to IPVA as a child has been comprehensively demonstrated to impact on life-course mental health outcomes and increases their risk of perpetrating IPVA or being victims themselves in adulthood [10–12].

Concerns about IPVA perpetration by serving and ex-serving military personnel in both the UK and the US are often reported in the media and within military and criminal justice circles [13–17]. Recent findings suggest IPVA perpetration in international military populations to be prevalent, and indeed, it has been shown to be more prevalent among UK military personnel than in the civilian population [18, 19]. There is mounting evidence that the post-deployment period is a time of higher risk of violence by military personnel [20, 21]. However, data on IPVA perpetration by UK military personnel in the post-deployment period are lacking. UK research into family violence among UK military personnel found that 3.6% reported hitting a family member in the weeks following return from deployment in Iraq/Afghanistan [22]; however, this study did not have data on partner violence.

Identifying the factors associated with IPVA perpetration has been a major research priority in an attempt to highlight targets for violence and harm reduction. A broad range of factors have been identified that increase the risk of being a perpetrator of IPVA in civilian populations, including male gender (for sexual and more severe forms of physical IPVA at least), younger age, low household income, low social support and family conflict [23], history of child abuse [24], and mental health or behavioural problems, including posttraumatic stress disorder (PTSD), and substance misuse [25–31].

There are reasons to suspect that there may be factors associated with relationship conflict and IPVA perpetration which are specific to military populations. In recognition of these potential differences, the UK Ministry of Defence have published their own domestic abuse strategy [32]. The demands of military life, which often requires frequent relocation and family separation, can add to relationship stress and create a context in which conflict and abuse may be more likely to occur [33–36]. The period following return from operational deployment has been identified in qualitative research as a time of heightened relationship conflict [34, 36, 37]. UK research has found that among a large sample of UK personnel returned from deployment in Iraq and Afghanistan, combat exposure was predictive of both general and family directed violence, but these studies did not measure partner violence [20–22, 38]. Findings from studies exploring the association between deployment and combat exposure or deployment-related trauma and IPVA have not been consistent [19, 39–41]. Post-deployment mental health problems, such as PTSD, and alcohol misuse have been found to be risk factors for partner violence in international studies [39, 41, 42]. These associations have been found for stranger and family directed violence perpetration among UK military personnel following return from deployment [20–22]. However, there remains a need for research examining the association between post-deployment mental health and behavioural difficulties and IPVA in the UK.

There is a lack of research into intimate relationships and partner violence in the UK military, in particular in the weeks following return from deployment. A better understanding of risk factors for post-deployment relationship conflict and partner violence in UK military personnel is needed to inform IPVA prevention, identification and management strategies as well as IPVA perpetrator programmes. To this end, this study aimed to use questionnaire data from a large sample of serving and ex-serving UK military personnel who deployed to Iraq or Afghanistan to estimate the prevalence of relationship conflict and partner violence perpetration in the weeks following return from deployment. The impact of role on deployment and exposure to deployment-related trauma will be examined, as well as the association of mental health and behavioural problems, such as PTSD, common mental disorder, difficulties with anger management and alcohol misuse, with these outcomes.

## Methods

### Study design and participants

These data were drawn from Phase 3 of data collection in an ongoing cohort study of the impact of operational deployment to Iraq/Afghanistan on the health and well-being of UK military personnel [43]. The cohort study commenced in June 2004 at the outset of the Iraq war and compared the health of two randomly selected samples: individuals who had deployed to the initial ground combat phase at the start of the conflict in Iraq (termed Op TELIC) were compared with individuals who were serving but who at that time had not deployed to Iraq (termed the Era group). At Phase 1, 10,272 participants were recruited between June 2004 and March 2006 (59% response rate) [44]. Of the Phase 1 participants, 6429 (56%) of the 9355 individuals who had consented to further contact completed Phase 2 of the study, which took place between November 2007 and September 2009 [45]. By Phase 2 of data collection, a second major deployment to Afghanistan (termed Op HERRICK) had commenced, and therefore, two additional samples were needed. A random sample of personnel deployed to Afghanistan between April 2006 and April 2007 along with a new sample of trained personnel who had joined service since April 2003 (the Phase 2 replenishment sample). A total of 9990 (56%) personnel responded at Phase 2 of the study.

Data collection for Phase 3 took place between October 2014 and December 2016. Phase 3 re-contacted participants who consented to further contact during Phase 1, or 2. The follow-up sample comprised 12,280 individuals; 10,148 regular and 2132 reserve personnel. A replenishment sample of trained regular and reserve personnel who joined the military on or after 1st August 2009 and were in service on the 31st March 2013 was also selected for sampling. This Phase 3 replenishment sample comprised 8581 individuals, 6915 regular and 1666 reserve personnel. Full details of the sampling and response rates have been reported previously [43]. This study utilised data from all those who responded to Phase 3 of the cohort study [43] and who had deployed to Iraq or Afghanistan (*n* = 5437). Those who responded ‘Not applicable’ to both relationship conflict or IPV questions (*n* = 825, suggestive of them not being in a relationship) and those who did not respond at all (*n* = 46) were excluded. The final sample consisted of 4,566 military personnel.

### Measures

Participation in Phase 3 of the study involved completing a self-administered questionnaire available both online and in hard copy. At the time of completing the questionnaire, participants may have returned from deployment in Iraq or Afghanistan between 4 months and 13 years, 11 months previously. Some may have left the military prior to participating in the study.

#### Socio-demographic and background characteristics

We examined data on sex, age at most recent deployment (under 25; 25–29; 30–34; 35–39; 40–44; 45 and over), relationship status (relationship; single/ex-relationship), and level of education (no qualification/O level equivalent, or A-level/degree level). We also asked 16 true/false questions about participants’ experiences (both adverse and protective) during childhood (Cronbach’s *α* = 0.751) [46]. Endorsed items were summed to create a vulnerability count: 0–2 (low); 3–5 (moderate); and 6 or more (high).

#### Military characteristics

We collected data on serving status (discharged; serving); service type (regular, reserve); service branch (Royal Navy, Army, Royal Air Force (RAF)); rank (Officer, non-commissioned officer (NCO), Other rank); deployment to Iraq and/or Afghanistan (Telic; Herrick); role on most recent deployment (non-combat; combat); and military trauma during most recent deployment—a cumulative score derived from endorsing a traumatic experience (13 in total) and the number of times it was experienced. Scores ranged from 0 to 52 (median = 5, IQR 2–12, *α* = 0.995), and were categorised into: 0, none; 1–5, mild; 6–12, moderate; and 13 or over, severe.

#### Mental health factors

Health questions enquired about symptoms of common mental disorder (CMD), measured using the 12-item General Health Questionnaire (GHQ-12; *α* = 0.960) [47]; probable posttraumatic stress disorder (PTSD), measured using the 17-item National Centre for PTSD Checklist (PCL-5; *α* = 0.963) [48]; alcohol use, measured using the 10-item World Health Organization Alcohol Use Disorders Identification Test (AUDIT; *α* = 0.0.779) [49]. Binary outcome variables were defined using the following cutoff scores for caseness: 4 or more for the GHQ-12 (scores range from 0 to 36) [50], 50 or more for the PCL-5 (scores range from 17 to 85) [48], and 16 or more for the AUDIT (scores range from 0 to 40) [51] (usually defined as hazardous use that is also harmful to health, which we have termed alcohol misuse). Difficulties with anger management were measured using a score of 12 or above on the dimensions of anger reactions (DAR; *α* = 0.902) [52].

#### IPVA outcomes

Questions concerning relationship conflict or IPVA on return from most recent deployment were asked as part of a series of questions on homecoming experiences with the common stem ‘In the weeks after I came home…’, (i) ‘I argued with my spouse or partner’, or (ii) ‘I was physically violent towards my spouse or partner’. These were divided into two IPVA outcomes for analysis: arguing with partner and being physically violent towards partner.

### Analyses

After running descriptive statistics on the whole sample, we conducted a series of univariate and multivariable logistic regression analyses for our male sample only (*n* = 4146) using Stata 16 [53]. Given what is known about gender differences in behaviours within relationships, ideally the analyses would have been stratified by gender. However, the number of females (and outcomes in females) was too low to allow reliable interpretation of the regression models. Therefore, results in males only have been reported.

First, logistic regression analyses were used to examine the bivariate associations between each of the two IPVA outcomes and the socio-demographic, military, and mental health factors in turn. Second, multivariate logistic regression analyses were conducted to examine the independent associations between each of the two IPVA outcomes and the socio-demographic variables. Finally, any socio-demographic variable that was independently associated with each IPVA outcome was retained as a covariate in subsequent multivariable logistic regression models examining the independent associations between each of the IPVA outcomes and each military and mental health factor. Given the potential for responder bias associated with the time since deployment, we conducted sensitivity analyses repeating our multivariate analyses with this variable as an additional covariate. To account for sampling and response rates [43], all analysis estimates were weighted using Stata’s survey function [53]. Prevalence estimates are reported as weighted proportions and 95% confidence intervals (CI); results from the univariate analyses are reported as odds ratios (OR) with 95% CI; and the results from the multivariate analyses are reported as adjusted ORs (aOR) with 95% CI.

## Results

### Description of the sample

The sample consisted of 4566 military personnel who had deployed to Telic and/or Herrick at any time since April 2003, were in a relationship following their return from their most recent deployment and who responded to at least one of the relationship conflict and IPVA questions (see Table 1). The majority of the sample was male (92.92%), aged under 34 at last deployment (57.31%; median = 34, IQR = 28–40), and educated to at least A-level standard (or equivalent; 67.66%). In terms of military characteristics, most were regular personnel (92.60%), in the Army (70.31%), and non-commissioned officers (NCO; 67.38%). A little over half of the sample (50.99%) were currently serving personnel.

**Table 1:** Characteristics of the sample (*N* = 4566)

Characteristic	*N* (unweighted)^a^	Weighted % (95% CI)
Sex		
Female	420	7.08 (6.31–7.93)
Male	4146	92.92 (92.07–93.69)
Age		
Under 25	720	18.47 (17.02–20.01)
25–29	849	19.39 (17.98–20.88)
30–34	907	19.45 (18.08–20.90)
35–39	1004	22.85 (21.36–24.41)
40–44	632	12.31 (11.24–13.47)
45 and over	454	7.53 (6.74–8.41)
Relationship status		
Relationship	3978	88.85 (87.66–89.94)
Single or ex-relationship	523	11.15 (10.06–12.34)
Education level		
No qual or O level	1347	32.34 (30.64–34.09)
A-level or degree	3196	67.66 (65.91–69.36)
Serving status		
Discharged	1816	49.01 (47.20–50.82)
Serving	2750	50.99 (49.18–52.80)
Status		
Regular	3844	92.60 (91.88–93.26)
Reserve	722	7.40 (6.74–8.12)
Service		
Naval	485	10.60 (9.55–11.74)
Army	3143	70.31 (68.67–71.90)
RAF	938	19.09 (17.76–20.50)
Rank		
Officer	1341	20.85 (19.61–22.15)
NCO	2749	67.38 (65.73–68.98)
Other	476	11.77 (10.57–13.08)
Deployment		
Telic	1617	36.82 (35.06–38.61)
Herrick	2949	63.18 (61.39–64.94)

^a^Numbers may not add up to total *N* due to missing data

### Prevalence of self-reported IPVA on return from deployment

The prevalence of self-reported IPVA on return from deployment is shown in Table 2. There was a high prevalence of self-reported arguments with spouses/partners in the weeks following return from deployment (34.66%, 95% CI 32.93–36.42) and males were significantly more likely than females to report this behaviour (35.37%, 95% CI 33.55–37.23 and 25.33%, 95% CI 20.74–30.54, respectively). A lower prevalence of physical violence towards spouses/partners was reported (3.42%, 95% CI 2.77–4.21), and males and females were just as likely to report this behaviour (3.46%, 95% CI 2.77–4.30 and 2.92%, 95% CI 1.57–5.38, respectively).

**Table 2:** Self-reported intimate partner violence by sex (*N* = 4566)

Variable	*n*/*N* (unweighted)*	Weighted % (95% CI)
Argued with spouse/partner		
Female	109/420	25.33 (20.74–30.54)
Male	1386/4137	35.37 (33.55–37.23)
Overall	1495/4557	34.66 (32.93–36.42)
Physically violent towards spouse/partner		
Female	12/418	2.92 (1.57–5.38)
Male	112/4132	3.46 (2.77–4.30)
Overall	124/4550	3.42 (2.77–4.21)

^a^Numbers may not add up to total *N* due to missing data

### Socio-demographic, military and pre-enlistment factors associated with post-deployment IPVA

In our male sample, participants aged 40 and over had reduced odds of arguing with a spouse/partner, compared to those aged under 25 (45 and over vs. under 25, aOR = 0.43, 95% CI 0.29–0.64; 40–44 vs under 25, aOR = 0.70, 95% CI 0.50–0.99; see Table 3). Participants no longer serving in the military at the time of completing the questionnaire were more likely to report both arguing with a spouse/partner (aOR = 1.24, 95% CI 1.04–1.48) and being violent towards a spouse/partner post-deployment (aOR = 2.01, 95% CI 1.21–3.34) compared to participants still serving in the military. Those serving in the RAF were less likely to report both arguing with (aOR = 0.77, 95% CI 0.62–0.97), and being violent towards (aOR = 0.16, 95% CI 0.06–0.45) a spouse/partner on return from deployment compared to those serving in the Army. Those who reported higher levels of childhood adversity were more likely to report both arguing with (high- and moderate- vs. low-adversity, aOR = 2.54, 95% CI 2.01–3.21 and aOR = 1.43, 95% CI 1.16–1.78, respectively), and being violent towards (high- vs. low-adversity, aOR = 3.63, 95% CI 1.65–7.95), a spouse/partner on return from deployment compared to those with low levels of childhood adversity.

**Table 3:** Socio-demographic, military and pre-enlistment factors associated with post-deployment intimate partner violence in our male sample (*N* = 4146)

Variable	Argued with spouse or partner			Violent towards spouse or partner		
	*n*/*N* (%^a^)	OR (95% CI)	aOR^b^ (95% CI)	*n*/*N* (%^a^)	OR (95% CI)	aOR^b^ (95% CI)
Age						
Under 25	235/633 (41.25)	1	1	28/632 (5.10)	1	1
25–29	256/746 (37.45)	0.85 (0.65–1.12)	0.87 (0.65–1.18)	24/744 (5.04)	0.99 (0.51–1.90)	1.45 (0.77–2.73)
30–34	280/796 (35.95)	0.80 (0.61–1.04)	0.96 (0.71–1.30)	17/797 (2.68)	0.51 (0.25–1.06)	0.96 (0.45–2.02)
35–39	335/938 (36.08)	0.80 (0.62–1.04)	0.85 (0.63–1.15)	18/934 (2.16)	0.41 (0.20–0.84)*	0.58 (0.27–1.22)
40–44	180/598 (30.74)	0.63 (0.47–0.85)**	0.70 (0.50–0.99)*	15/598 (2.59)	0.49 (0.22–1.11)	0.93 (0.38–2.29)
45 and over	100/426 (20.47)	0.37 (0.26–0.51)***	0.43 (0.29–0.64)***	10/427 (2.93)	0.56 (0.25–1.28)	1.47 (0.57–3.80)
Education						
No qual or O level	439/1252 (36.73)	1.09 (0.91–1.29)	0.98 (0.81–1.19)	48/1252 (3.85)	1.20 (0.76–1.90)	0.90 (0.55–1.47)
A-level or degree	942/2864 (34.82)	1	1	62/2859 (3.22)	1	1
Serving status						
Veteran (ex-serving)	589/1641 (37.77)	1.23 (1.05–1.44)*	1.24 (1.04–1.48)*	65/1639 (4.61)	2.01 (1.29–3.14)**	2.01 (1.21–3.34)**
Serving	797/2496 (33.05)	1	1	47/2493 (2.34)	1	1
Status						
Regular	1166/3517 (35.32)	1	1	90/3513 (3.37)	1	1
Reserve	220/620 (36.08)	1.03 (0.82–1.30)	1.10 (0.86–1.41)	22/619 (4.65)	1.40 (0.79–2.48)	1.04 (0.54–1.99)
Service						
Naval services	150/445 (34.33)	0.87 (0.67–1.12)	1.01 (0.77–1.32)	14/447 (3.73)	0.89 (0.46–1.72)	1.02 (0.50–2.08)
Army	986/2851 (37.58)	1	1	93/2848 (4.16)	1	1
RAF	250/841 (27.64)	0.63 (0.52–0.78)***	0.77 (0.62–0.97)*	5/837 (0.62)	0.14 (0.05–0.38)***	0.16 (0.06–0.45)***
Rank						
Officer	327/1174 (28.47)	0.70 (0.58–0.84)***	0.94 (0.76–1.17)	15/1174 (1.58)	0.46 (0.24–0.88)*	0.50 (0.22–1.10)
NCO	886/2514 (36.34)	1	1	71/2509 (3.40)	1	1
Other rank	173/449 (41.45)	1.24 (0.96–1.61)	1.04 (0.77–1.41)	26/449 (6.91)	2.11 (1.21–3.67)**	1.79 (0.98–3.25)^c^
Childhood adversity						
Low (0–2)	264/1062 (25.34)	1	1	14/1061 (1.64)	1	1
Moderate (3–5)	585/1792 (33.31)	1.47 (1.19–1.82)***	1.43 (1.16–1.78)**	39/1789 (2.66)	1.64 (0.77–3.51)	1.60 (0.72–3.53)
High (> = 6)	455/1051 (47.41)	2.66 (2.11–3.34)***	2.54 (2.01–3.21)***	53/1050 (6.46)	4.14 (1.96–8.71)***	3.63 (1.65–7.95)

^a^Weighted

^b^aOR (adjusted Odds Ratio) adjusted for age, education, serving status, status, service, rank and childhood adversity

^c^*p* = 0.056 as this was close to significance, rank was included as a confounder in the adjusted models for violent towards spouse/partner

^*^p < 0.05

**p < 0.01

***p < 0.001

### Deployment factors associated with post-deployment IPVA

Having been deployed in a combat role compared to a non-combat role was independently associated with arguing with a spouse/partner on return from deployment (aOR = 1.40, 95% CI 1.18–1.66; see Table 4). Increasing levels of military-related trauma were associated with arguing with a spouse/partner post-deployment compared to no military-related trauma (severe, moderate, and low vs. none, aOR = 3.29, 95% CI 2.46–4.42, aOR = 2.39, 95% CI 1.79–3.18, and aOR = 1.74, 95% CI 1.31–2.31, respectively; see Table 4). Severe military trauma exposure was also independently associated with violence towards a spouse/partner on return from deployment compared to no military-related trauma (severe vs. none, aOR = 2.30, 95% CI 1.11–4.78).

**Table 4:** Deployment factors associated with post-deployment intimate partner violence in our male sample (N = 4146)

Variable	Argued with spouse or partner			Violent towards spouse or partner		
	*n*/*N* (%^a^)	OR (95% CI)	aOR^b^ (95% CI)	*n*/*N* (%^a^)	OR (95% CI)	aOR^c^ (95% CI)
Deployment						
Non-combat	708/2347 (30.64)	1	1	59/2343 (2.93)	1	1
Combat	677/1783 (41.13)	1.58 (1.35–1.86)***	1.40 (1.18–1.66)***	53/1783 (4.09)	1.41 (0.90–2.22)	0.97 (0.61–1.53)
Military trauma						
None	142/766 (18.97)	1	1	13/766 (1.81)	1	1
Mild	313/1092 (30.55)	1.88 (1.43–2.47)***	1.74 (1.31–2.31)***	21/1092 (2.09)	1.16 (0.51–2.62)	1.06 (0.46–2.44)
Moderate	336/910 (37.63)	2.58 (1.95–3.40)***	2.39 (1.79–3.18)***	16/910 (1.94)	1.07 (0.45–2.54)	0.87 (0.36–2.10)
Severe	419/901 (48.74)	4.06 (3.08–5.35)***	3.29 (2.46–4.42)***	46/899 (6.60)	3.83 (1.83–8.02)***	2.30 (1.11–4.78)*

^a^Weighted

^b^aOR (adjusted Odds Ratio) adjusted for age, serving status, service and childhood adversity

^c^aOR adjusted for serving status, service, rank and childhood adversity

^*^p < 0.05

***p < 0.001

### Mental health factors associated with post-deployment IPVA

Arguing with a spouse/partner in the weeks following return from deployment was independently and strongly associated with probable PTSD (aOR = 5.71, 95% CI 3.85–8.47), alcohol misuse (aOR = 2.40, 95% CI 1.84–3.12), probable CMD (aOR = 3.04, 95% CI 2.44–3.78) and increased difficulties with anger management (aOR = 3.69, 95% CI 2.88–4.73), see Table 5. Being violent towards a spouse/partner following deployment was also independently and strongly associated with probable PTSD (aOR = 4.82, 95% CI 2.72–8.52, alcohol misuse (aOR = 2.32, 95% CI 1.36–3.97), probable CMD (aOR = 2.79, 95% CI 1.70–4.57), and increased difficulties with anger management (aOR = 5.72, 95% CI 3.43–9.54).

**Table 5:** Mental health factors associated with post-deployment intimate partner violence in our male sample (*N* = 4146)

Variable	Argued with spouse or partner			Violent towards spouse or partner		
	*n*/*N* (%^a^)	OR (95% CI)	aOR^b^ (95% CI)	*n*/*N* (%)	OR (95% CI)	aOR^c^ (95% CI)
Probable PTSD						
No	1198/3849 (32.62)	1	1	77/3845 (2.50)	1	1
Yes	176/232 (76.54)	6.74 (4.61–9.86)***	5.71 (3.85–8.47)***	34/230 (16.89)	7.92 (4.75–13.22)***	4.82 (2.72–8.52)***
Alcohol misuse						
No	1130/3629 (32.55)	1	1	84/3623 (2.91)	1	1
Yes	233/441 (56.37)	2.68 (2.09–3.43)***	2.40 (1.84–3.12)***	28/442 (7.88)	2.85 (1.69–4.81)***	2.32 (1.36–3.97)**
Common mental disorders						
No	895/3207 (29.20)	1	1	56/3206 (2.21)	1	1
Yes	479/884 (57.88)	3.39 (2.76–4.17)***	3.04 (2.44–3.78)***	54/880 (7.78)	3.93 (2.47–6.26)***	2.79 (1.70–4.57)***
Anger score						
0–11	986/3452 (29.69)	1	1	47/3448 (1.71)	1	1
12 +	348/553 (65.08)	4.41 (3.48–5.59)***	3.69 (2.88–4.73)***	59/552 (12.72)	8.37 (5.18–13.52)***	5.72 (3.43–9.54)***

^a^Weighted

^b^aOR (adjusted Odds Ratio) adjusted for age, serving status, service and childhood adversity

^c^aOR adjusted for serving status, service, rank and childhood adversity

**p < 0.01

***p < 0.001

### Sensitivity analyses

In our sensitivity analyses, we found that the association between serving status and both arguing with or being physically violent towards a spouse/partner upon return from deployment was no longer significant. This was likely due to the high collinearity between serving status and time since deployment. All other independent associations persisted after controlling for the time since the most recent deployment.

## Discussion

This study found that among UK military personnel who were deployed to Iraq/Afghanistan between 2002 and 2016, relationship conflict (arguing) was highly prevalent (34.7%) in the weeks following return from deployment. This aligns with recent findings that the post-deployment period is perceived as a period of higher risk of relationship difficulties as well as abusive behaviours by military personnel [34, 36]. Physical intimate partner violence (IPV) was less commonly reported (3.4%) than arguing, but its prevalence adds to the growing evidence for the occurrence of general, family and now partner directed violence post-deployment among UK military personnel [22]. Our findings also provide valuable insight into factors associated with increased relationship conflict or physical IPV in the post-deployment period and identify groups most at risk of engaging in this behaviour. This may be helpful in understanding the higher prevalence of IPVA perpetration in UK military personnel compared to the general population in the UK [19, 20], particularly providing further support for the role of exposure to deployment-related trauma and mental health and alcohol misuse difficulties.

Gender differences were not observed in the perpetration of physical IPV in the post-deployment period, which is in keeping with some research findings of similar rates of physical IPV perpetration among males and females in both military and civilian populations [19, 24]. However, this finding is not consistent in other research studies which have found both higher prevalence of physical IPV perpetration among males compared to females [18] and alternately females compared to males [54, 55]. Males were more likely to report arguing with their intimate partners/spouses post-deployment than female personnel, which echoes recent research among UK military personnel which found that males were significantly more likely to report perpetration of non-physical forms of IPVA (emotional or psychological abuse) than females [19]. In contrast with previous general violence research among UK military personnel [20, 21, 56, 57], we did not find a clear socio-demographic profile for those who reported relationship conflict or physical IPV. Contrasting with previous research, younger age was independently associated with arguing with an intimate partner following deployment but not physical IPV [14, 42, 58], and no further socio-demographic factors were found to be associated with relationship conflict or IPV. However, the association between childhood adversity and both relationship conflict and physical IPV in the post-deployment period in this study adds to findings from wider military research, which have highlighted the role of early life adversity in IPVA perpetration [19, 40, 42, 58], and identified it as an important factor to be considered in risk assessments of future IPVA.

A number of military characteristics have been found in repeated studies to be associated with violence post-deployment by UK military personnel, including serving in the Army, being of lower rank, engagement status and having left service [20, 22]. By contrast, IPVA perpetration among UK military personnel has only been shown to be significantly more likely among Army and Royal Navy personnel, compared to RAF [19]. In keeping with the aforementioned research, the current study found a higher risk of relationship conflict and physical IPV post-deployment for Army compared to RAF personnel (risk for Royal Navy personnel was similar to Army personnel). In addition, veteran personnel (ex-serving) were significantly more likely to report both arguing with their partner and perpetration of physical IPV post-deployment, which adds to findings from international studies in to military IPVA perpetration [18]. However, these associations were no longer significant after adjusting for time since most recent deployment. This is suggestive of issues relating to response bias and disclosure, whereby participants may be more likely to disclose relationship conflict and IPV in the post-deployment period once they have left the military. Qualitative research into help-seeking for IPVA among military personnel has identified a number of barriers to reporting while still serving [59], including a perception that help-seeking could ‘let the side down’ and impact colleagues, result in military personnel appearing weak and not able to cope, or negatively impact their career and opportunities for promotions. Services working with military personnel must be mindful of these challenges relating to bias and disclosure for IPVA identification, risk assessment and management.

Deployment-related variables and mental health and alcohol misuse problems were found to be key factors associated with post-deployment relationship conflict and IPV. Adding to mounting evidence for the link between deployment-related trauma and IPVA perpetration [19, 39, 41, 60], intensity of exposure to trauma while on deployment was associated with increased risk of relationship conflict and IPV perpetration in the weeks following deployment. Role on deployment was only found to be associated with relationship conflict post-deployment and not physical violence. This contrasts with wider military violence literature, which found combat role to be a significant factor in post-deployment family violence [22]. The subjective experience of trauma on deployment is likely to be a more sensitive measure of deployment experience than role on deployment, which may not accurately capture trauma or combat exposure.

Probable mental health difficulties and alcohol misuse were strongly and independently associated with relationship conflict and physical IPV perpetration in the weeks following deployment, in keeping with a large body of research linking mental health difficulties and alcohol misuse with relationship conflict and IPVA [19, 39, 42, 61]. Our findings point to the role that PTSD, especially deployment-related, may play in relationship conflict and IPV perpetration post-deployment. However, given the cross-sectional nature of the data, the direction of that association could not be established in this study. Recent qualitative research by our group has facilitated better understanding of the complexity and nuances of the association between deployment, mental health difficulties and IPVA perpetration [34, 36]. That research highlighted the significance of separations and difficulties re-adjusting to family life post-deployment in creating context for relationship tensions and conflict. In addition, mental health and psychological difficulties were perceived by both military personnel [36] and civilian victim-survivors of abusive relationships with military personnel [34] to contribute to relationship conflict and IPVA post-deployment. Difficulties adjusting post-deployment, and mental health difficulties in particular, were perceived to amplify other influences of military culture and socialisation, which were observed to spill over into the home and affect relationships and risk of IPVA [36], such as a need for order and control and aggressive communication styles.

This study provides much needed insight into factors associated with relationship conflict and physical IPV perpetration in the weeks following return from military deployment to Iraq or Afghanistan. However, despite a large sample size, low numbers of female personnel precluded our ability to include gender in regression analyses, which were run using our male sample only. We must acknowledge that participants may have under-reported their relationship conflict and physical IPV perpetration in this study, as in other population studies of IPVA [62, 63], and that findings are representative of those who reported being in a relationship following their most recent deployment and endorsed relationship conflict or physical IPV perpetration in the questionnaire. In addition, this study does not capture the potential bidirectional nature of IPV, which has been shown to be common in military communities [19, 40, 64]. Although not the focus of the study, information on participants’ ethnicity or sexual orientation and sexual violence post-deployment was not collected. Further research should include measures of frequency and impact of IPV, explore sexual IPV and bidirectional abuse in more depth, and the role of ethnicity and sexual orientation, which may differentially impact risk of IPVA [39, 42, 65]. Longitudinal design is also needed to robustly examine the role of mental health difficulties in the perpetration of IPVA.

This study importantly highlights the risk of relationship conflict and physical IPV upon return from deployment within military relationships and identifies factors which are associated with this risk. Although the UK military is not currently engaged in regular operational deployments, the impact of past deployments and deployment-related trauma have been suggested to have longer lasting effects and contribute to IPVA beyond the peri-deployment period [34, 36]. As such, services providing health or welfare support to serving and ex-serving personnel and their families must consider history of deployment, and particularly trauma experienced on deployment, in their risk assessments to improve identification and management of IPVA in military communities. These findings also suggest that any strategy to improve identification, management and prevention of IPVA must involve mental health services. Integrated referral pathways and a widespread uplift in training to increase awareness and understanding of IPVA, as well as the potential impact of deployment-related trauma and mental health difficulties, would support such strategies.

## Supplementary Information

Below is the link to the electronic supplementary material.

Supplementary file1 (DOCX 20 kb)

Supplementary file2 (DOCX 18 kb)
