# Supplementary material for: Relationship conflict and partner violence by UK military personnel following return from deployment in Iraq and Afghanistan
Source: Soc Psychiatry Psychiatr Epidemiol. 2022 Jun 5;57(9):1795–805. doi: 10.1007/s00127-022-02317-8 (PMC9167453; doi:10.1007/s00127-022-02317-8)
Supplement: Supplementary file 1 — Supplementary file1 (DOCX 20 kb) [file 127_2022_2317_MOESM1_ESM.docx]

Supplementary Table 1:

|  |  | Argued with partner | Violent towards partner |
| --- | --- | --- | --- |
|  |  | aOR ^a^ (95% CI) | aOR ^b^ (95% CI) |
| Age | |  |  |
|  | Under 25 | -Reference- | -Reference- |
|  | 25-29 | 0.870 (0.645-1.174) | 1.626 (0.860-3.076) |
|  | 30-34 | 0.960 (0.708-1.301) | 1.036 (0.482-2.228) |
|  | 35-39 | 0.849 (0.627-1.150) | 0.620 (0.292-1.318) |
|  | 40-44 | 0.702 (0.497-0.992)* | 1.148 (0.461-2.858) |
|  | 45 & Over | 0.431 (0.288-0.644)*** | 1.886 (0.714-4.982) |
| Education | |  |  |
|  | No qual or O level | 0.985 (0.813-1.194) | 0.896 (0.549-1.463) |
|  | A level or degree | -Reference- | -Reference- |
| Serving status | |  |  |
|  | Discharged | 1.251 (1.014-1.542)* | 1.241 (0.690-2.232) |
|  | Serving | -Reference- | -Reference- |
| Status | |  |  |
|  | Regular | -Reference- | -Reference- |
|  | Reserve | 1.103 (0.856-1.421) | 0.833 (0.429-1.618) |
| Service | |  |  |
|  | Naval Services | 1.012 (0.773-1.324) | 0.891 (0.426-1.863) |
|  | Army | -Reference- | -Reference- |
|  | RAF | 0.775 (0.621-0.967)* | 0.157 (0.055-0.448)*** |
| Rank | |  |  |
|  | Officer | 0.944 (0.764-1.167) | 0.458 (0.206-1.018) |
|  | NCO | -Reference- | -Reference- |
|  | Other rank | 1.043 (0.769-1.413) | 2.000 (1.078-3.711)* |
| Childhood Adversity | |  |  |
|  | 0-2 | -Reference- | -Reference- |
|  | 3-5 | 1.435 (1.156-1.780)** | 1.578 (0.712-3.501) |
|  | >=6 | 2.539 (2.006-3.213)*** | 3.741 (1.695-8.253)** |
|  |  |  |  |
| Days since deployment | | 1.000 (1.000-1.000) | 1.000 (1.000-1.001)*** |
| ^a^ aOR (adjusted Odds Ratio) adjusted for age, education, serving status, status, service, rank, childhood adversity, and days since deployment  * p<0.05, **p<0.01 ***p<0.001 | | | |
