# Supplementary material for: Relationship conflict and partner violence by UK military personnel following return from deployment in Iraq and Afghanistan
Source: Soc Psychiatry Psychiatr Epidemiol. 2022 Jun 5;57(9):1795–805. doi: 10.1007/s00127-022-02317-8 (PMC9167453; doi:10.1007/s00127-022-02317-8)
Supplement: Supplementary file 2 — Supplementary file2 (DOCX 18 kb) [file 127_2022_2317_MOESM2_ESM.docx]

Supplementary Table 2:

|  |  | Argued with partner | Violent towards partner |
| --- | --- | --- | --- |
|  |  | aOR (95% CI) | aOR (95% CI) |
| Deployment | |  |  |
|  | Non-combat | -Reference- | -Reference- |
|  | Combat | 1.40 (1.18-1.66)*** | 1.00 (0.62-1.59) |
| Military trauma | |  |  |
|  | None | -Reference- | -Reference- |
|  | Mild | 1.73 (1.31-2.30)*** | 1.07 (0.46-2.46) |
|  | Moderate | 2.40 (1.80-3.20)*** | 0.81 (0.33-1.99) |
|  | Severe | 3.29 (2.46-4.41)*** | 2.28 (1.09-4.77)* |
| Probable PTSD | |  |  |
|  | No | -Reference- | -Reference- |
|  | Yes | 5.71 (3.85-8.47)*** | 4.72 (2.67-8.35)*** |
| Alcohol misuse | |  |  |
|  | No | -Reference- | -Reference- |
|  | Yes | 2.40 (1.84-3.13)*** | 2.46 (1.44-4.20)** |
| Common mental disorders | |  |  |
|  | No | -Reference- | -Reference- |
|  | Yes | 3.05 (2.44-3.80)*** | 2.68 (1.64-4.38)*** |
| Anger score | |  |  |
|  | 0-11 | -Reference- | -Reference- |
|  | 12+ | 3.69 (2.88-4.73)*** | 5.97 (3.58-9.93)*** |
| ^a^ aOR adjusted for age, serving status, service, childhood adversity, and days since deployment  ^b^ aOR adjusted for service, rank, childhood adversity, and days since deployment  * p<0.05, **p<0.01 ***p<0.001 | | | |
